# The potential impact of iron supply on the development of starved *Enterococcus faecalis* biofilm by modulating the liberation of extracellular DNA

**DOI:** 10.3389/fmicb.2025.1526909

**Published:** 2025-05-07

**Authors:** Yuqi Zhen, Quzhen Baima, Shipeng Yang, Yu Cao, XiuPing Meng

**Affiliations:** Jilin Provincial Key Laboratory of Oral Biomedical Engineering, Hospital of Stomatology, Jilin University, Changchun, China

**Keywords:** *Enterococcus faecalis*, starvation, iron supply, biofilm, eDNA

## Abstract

*Enterococcus faecalis* (*E. faecalis*) is commonly associated with persistent periapical infections. Even after multiple courses of root canal therapy, the infection is difficult to eradicate due to its drug resistance and adaptability. However, root canal treatment will remove nutrients from the root canal and make the remaining *E. faecalis* near starvation. Iron is an essential element for the growth and metabolism of *E. faecalis*, but previous studies were mostly based on bacterial nutrient sufficient conditions. Therefore, in this study, the starvation state was used as the breakthrough point to explore the mechanism of iron on the biofilm formation of *E. faecalis*, so as to be more suitable for clinical practice. In this study, we first constructed a starving *E. faecalis* model. Subsequently, we found that iron supply promoted biofilm formation in starved *E. faecalis*, with more eDNA in the biofilm. Iron starvation induced by the iron competitive inhibitor gallium nitrate reduced biofilm formation but increased the proportion of eDNA. In contrast, high iron levels in the environment counteracted this inhibition of biofilm formation. Following DNase I treatment, both the eDNA content and viable bacteria within the biofilm of the iron-supply group exhibited a statistically significant reduction. These results suggest that iron supply may regulate the proliferation of active bacteria by regulating eDNA release, thereby promoting biofilm formation of starved *E. faecalis* and providing a new perspective on its survival strategy under stress.

## 1 Introduction

*Enterococcus faecalis* (*E. faecalis*) was one of the most commonly isolated bacteria in root canals of patients with persistent periapical disease, accounting for 45.8% of the total bacterial species isolated (Elashiry et al., 2023). For *E. faecalis* to be involved in the pathogenesis and maintenance of apical periodontitis, it must survive in the root filled canal where the nutrient supply is limited (Chen et al., 2017). *E. faecalis* has been reported to survive for up to 4 months in water and a limited phosphate or amino acid medium (Gutierrez et al., 2022). A capacity to endure starvation is a distinctive characteristic that might allow *E. faecalis* to survive until an opportunity for acquiring suitable nutrition becomes available. However, little is known about the nutrients in the root canal after root canal filling, and the nutrients needed to maintain the microflora may come from the fluid in the dentin tubule or the serum-like fluid in the periapical tissue. It has been found that the starved *E. faecalis* can recover and grow from the serum, thus playing a pathogenic role (Figdor et al., 2003).

Serum contains a variety of micronutrients (Vitale et al., 2019), of which iron is an essential micronutrient for bacterial survival. A large number of enzymes involved in respiration, nitrogen fixation and amino acid synthesis require iron to maintain structural stability and function (Lopez et al., 2012). Thus, during bacterial infections, both the host and bacteria compete for iron to thrive. In the human body, the majority of iron is sequestered within hemoglobin. Simultaneously, iron-binding proteins, along with antibodies and complement proteins in the body, work in concert to retain iron (Spiga et al., 2023). In response to the iron-starvation environment imposed by the host, bacteria have evolved a diverse array of mechanisms for iron uptake. Among these, the secretion of siderophores represent one of the most effective strategies. Siderophores, which are high-affinity ferric chelators, can complex with iron outside the cell and thereby mediate the process of iron uptake (Skaar, 2010). However, the capacity to synthesize siderophores is not universal among bacteria. Computer-based and transcriptomic studies have revealed that *E. faecalis* primarily facilitates the transmembrane transport of iron ions through the utilization of iron transporters (Brunson et al., 2022). Additionally, *E. faecalis* has the ability to internalize heme, enabling its degradation within the cell and the subsequent release of iron ions (Zhang et al., 2022). Interestingly, *E. faecalis* is highly tolerant of iron-restricted environments (Gaca and Lemos, 2019). It also has the ability to resist reactive oxygen-mediated oxidative stress under iron excess (Lopez et al., 2012). In response to changes in iron availability, *E. faecalis* evolved a complex system to regulate iron uptake and outflow, which provides advantages for its survival and pathogenicity (Ali et al., 2022).

*Enterococcus faecalis* in root canals mainly exists as a biofilm by adhering to root canal walls and forming communities that are 1,000 times more resistant to environmental pressure than isolated planktonic organisms (Ramakrishnan et al., 2022). Bacteria in biofilms are encased in a matrix composed of proteins, extracellular DNA (eDNA), and polysaccharide intercellular adhesion proteins (Xia et al., 2021). Among them, eDNA plays an important role in biofilm formation, facilitating horizontal gene transfer and being involved in bacterial twitch movement to promote biofilm expansion (Gloag et al., 2013; Sivalingam et al., 2020). When bacteria are starved, eDNA participates in forming biofilm bridges, enhancing the internal structure and communication of biofilms (Lei et al., 2019). During the development of *E. faecalis* biofilm, eDNA is released mainly through autolysis or “sibling killing” cell lysis (Schiopu et al., 2023). Thomas et al. (2009) demonstrated that two proteases, GelE and SprE, play crucial roles in finely regulating bacterial autolysis and eDNA release. SprE inhibits GelE – mediated lysis. Under normal circumstances, it restricts the degree of bacterial autolysis, ensuring that not all bacteria undergo autolysis to release eDNA. Once SprE is inactivated, the lysis rate surges, leading to increased eDNA release and enhanced biofilm development. Additionally, the expression and distribution of autolysins in bacteria are under strict regulation. Not all autolysins can be activated by GelE, which further confines the scope of eDNA release. As a result, only certain bacteria are capable of releasing eDNA through autolysis (Thomas et al., 2009).

The understanding of how iron influence *E. faecalis* virulence, alter it transcriptome, and how this pathogen regulates metal homeostasis has advanced significantly over the past few years (Keogh et al., 2016; Colomer-Winter et al., 2017; Govindarajan et al., 2022). However, current studies focused on iron metabolism in *E. faecalis* under nutrient-sufficient conditions, leaving gaps in understanding how iron supply influences biofilm formation under unique starvation conditions, especially the complex interplay between iron, eDNA release, and biofilm structural stability. In this study, we aimed to bridge this gap by using starved *E. faecalis* as a model to explore the relationship between iron supply and biofilm formation dependent on eDNA release, which enriched our understanding of microbial adaptation to extreme environments and helped to further elucidate the specific role of eDNA in biofilm structural stability and stress resistance.

## 2 Materials and methods

### 2.1 Bacterial strains, media, materials, and reagents

*Enterococcus faecalis* (ATCC 29212; Guangdong Provincial Key Laboratory of Microbiol Culture Collection and Application, Guangdong Institute of Microbiology, Guangzhou, China) was used in this study; 500 μL of bacteria (1 × 10^6^ CFU/mL) were added to 50 mL brain heart infusion (BHI) medium, which was further incubated without shaking at 37°C under anaerobic condition. Under the condition that no new nutrients were added, the bacterial suspension was collected at each time point at 0, 2, 4, 8, 12, 24, 48, 72 and 96 h, respectively, and its absorbance at 600 nm was measured. The growth curve of *E. faecalis* in BHI medium was obtained.

Disks manufactured from a medical-grade titanium alloy (14 mm in diameter and 1 mm thick; surface area: 1.54 cm^2^) (Depuy Synthes, Raynham, MA, United States) were used as the prosthetic material for biofilm adherence. Iron chloride hexahydrate (FeCl₃·6H₂O, 99% purity) and Gallium nitrate hydrate (Ga(NO₃)₃·xH₂O, 98% purity) were purchased from Aladdin Reagent (Shanghai, China). LIVE/DEAD^®^BacLight^TM^ bacterial viability kit was purchased from Sigma-Aldrich (St. Louis, MO, United States); Calcein AM and PI were purchased from Beyotime (Shanghai, China). Crystal violet staining solution and DNase I were purchased from Thermo Fisher Scientific (Cleveland, OH, United States).

### 2.2 Colony-forming unit (CFU) counting

Cell growth were monitored by the colony-forming unit (CFU) counting of the culturable bacteria on BHI agar plate as described by Portenier et al. (2005). In detail, the bacterial cells incubated for 48 h in BHI medium were collected. The pellets obtained by centrifugation were washed twice in 1 mL PBS. The cells were finally resuspended in PBS, and the viable count was adjusted to 1 × 10^6^ CFU/mL. We added 100 μL of bacteria at a final concentration of 1 × 10^6^ CFU/mL to each well of a 96-well microplate. Serial 2-fold dilutions of Fe salt, or Ga salt were then added to each well. Viable counts were determined by serial 6-fold dilutions on BHI plates incubated overnight in an incubator at 37°C.

### 2.3 Biofilm biomass assay

Bacterial cells incubated for 48 h in BHI medium were collected and adjusted to a uniform concentration of 1 × 10^8^ CFU/mL. In the absence of additional new nutrients, three replicate wells were set up for each group in a 96-well plate. PBS, Fe salt, Ga salt, or a 1:1 Fe-Ga mixed salt solution were added to each group. After 48 h of anaerobic cultivation, biofilms were formed. The liquid in the well plate was aspirated, and the wells were washed twice with PBS. Subsequently, 150 μL of crystal violet dye solution was added to each well and left at room temperature for 1 h. After thorough drying, 200 μL of dimethyl sulfoxide was added to each well to completely dissolve the crystal violet bound to the biofilm. To precisely measure the absorbance value for the quantitative assessment of biofilms, the solution was transferred to a new 96-well plate, and the absorbance value at 540 nm (A540) was measured using a microplate reader.

### 2.4 Scanning electron microscopy (SEM)

A 14-mm disk was placed in each well of a 24-well plate and 1 mL of *E. faecalis* bacterial solution incubated for 48 h in BHI medium was added. PBS, Fe salt, Ga salt, or a 1:1 Fe-Ga mixed salts solution were added to each group. After incubation for 48 h to form biofilms, the bacteria were washed twice with PBS and fixed with 2.5% glutaraldehyde for 2 h at 4°C. Further preparations included dehydration through a series of ethanol rinses (30, 50,70, 95, and 100%) and critical point drying with liquid CO_2_ before coating with gold (Hitachi E-1010, Ibaraki, Japan) with the thickness of approximately 20 nm. SEM of at least three randomly selected regions at different magnifications were obtained from each sample using a SEM operated at 20 kV (Hitachi S-3000N). Images presented in the following results were representative samples.

### 2.5 Confocal laser scanning microscopy (CLSM)

Confocal laser scanning microscopy (CLSM) was used to investigate the effect of iron supply on the biofilm structure of *E. faecalis*. At the beginning of the experiment, *E. faecalis* biofilms were constructed under different conditions. Before the images were obtained, the biofilm was stained according to the instructions using the LIVE/DEAD^®^BacLight^TM^ bacterial viability kit. Briefly, a working solution of fluorescent stains was prepared by adding 3 μL SYTO 9 stain and 3 μL PI stain to 1 mL filter sterilized water to prepare a working solution of fluorescent stain. Add 200 μL dye solution to each well and culture in room temperature darkness for 15 min. Rinse the sample with sterile saline to remove excess dye. The stained biofilm was detected by CLSM (Zeiss LSM880, Carl Zeiss AG, Oberkochen, Germany). SYTO 9 was excited by a 488 nm laser line to detect fluorescence emission in the range of 500 ~ 540 nm. PI was excited by a 561 nm laser line and its fluorescence emission was detected in the range of 600 ~ 695 nm. 3D side-view image acquisition was executed with ZEN 3.5 Blue Edition software (Carl Zeiss, Jena, Germany). Sequential 1-μm optical sections were systematically collected across the entire biofilm thickness using a × 63 oil immersion objective. The aligned optical slice calculation was then reconstructed into 3D volume rendering by maximum intensity projection (MIP) method. Biofilm thickness was finally measured using the Z-section measurement tool in ZEN, and three independent measurements were made for each sample.

### 2.6 eDNA staining and analysis

The biofilm cells and eDNA matrix components were stained with fluorescent dyes and observed with Fluorescence microscopy. Add 5 μL Calcein AM green fluorescent stain and 5 μL PI red fluorescent nucleic acid staining were prepared in sterile 0.9% NaCl, incubated at room temperature for 30 min in the dark and then washed twice with sterile 0.9% NaCl. Finally, biofilms were observed and imaged using a Fluorescence microscopy. Then, we used Image J software to conduct quantitative analysis of the observed biofilm images. After opening the images, a single channel was extracted and reversed, and the PI stained area in the biofilm showed black or gray. The threshold adjustment option of the Image J software is then used to select the areas occupied by PI staining in the biofilm. Make sure the threshold is adjusted to include all pixels that appear gray or black (not white). Then the average fluorescence Density of eDNA is Mean = Integrated Density/Area. Similarly, the Calcein-labeled active bacterial microclusters in the biofilm were quantified in the same way. In order to improve the comparability between red and green fluorescence intensities, we normalized the obtained data to obtain the relative fluorescence Density (RFD). To investigate the correlation between iron supply and the role of eDNA in promoting biofilm formation, the biofilms in the iron-supply group were treated with 28 U/mL DNase I for 1 h and 2 d respectively, and then analyzed.

### 2.7 Statistical analyses

All experiments were performed in triplicate. Statistical analyses were performed using GraphPad Prism (GraphPad Software, La Jolla, CA, United States). For each experiment, means ± standard deviations (SD) were calculated. Differences between means values were determined using one-way analysis of variance (ANOVA), with *p*-values < 0.05 considered statistically significantly.

## 3 Results

### 3.1 Establishment of starved *E. faecalis* model

**Figure 1:**
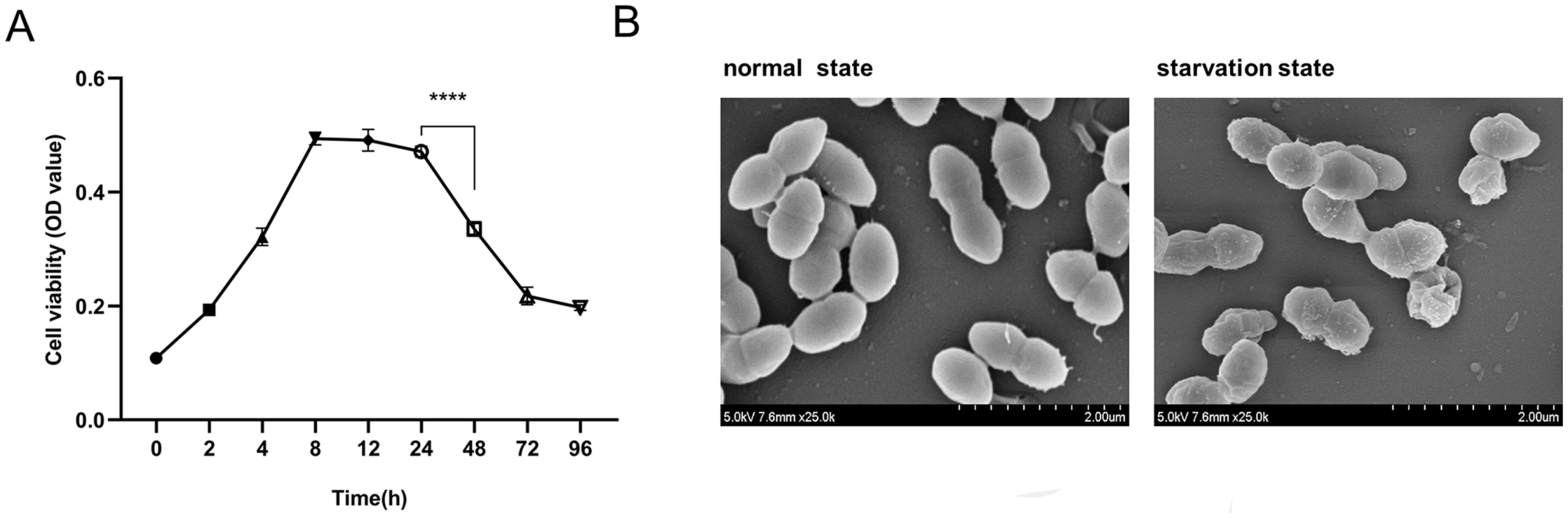
Establishment of a starvation state model of *Enterococcus faecalis*. **(A)** The growth curve of *E. faecalis* in BHI medium. Data were from three independent experiments. ****p < 0.0001. **(B)** SEM images depict the morphological characteristics of *E. faecalis* biofilms in its normal and starved states. Magnification: −25000×.

To establish an E. faecalis starvation model that accurately mimics the infectious state in root canals, E. faecalis was incubated in BHI culture at 37°C, and the optical density (OD) of bacterial suspension at 600 nm was measured over a period of 0–96 h to monitor bacteria growth. During the 0–8 h period, E. faecalis exhibited vigorous and rapid proliferation, characterized by typical logarithmic growth kinetics. The bacterial population reached its maximum level and subsequently entered a stationary phase during the following 8–24 h. The depletion of nutrients between 24 and 48 h resulted in a significant reduction in bacterial growth (p *< 0.05*), followed by stabilization (Figure 1A). To validate the establishment of a starvation model for E. faecalis, we employed SEM to observe the cultured E. faecalis in BHI medium after 8 and 48 h. After incubating in BHI culture medium for 8 h, the bacteria have smooth surfaces; After 48 h of incubation, the bacterial surface displays irregular wrinkling, but the overall integrity was still maintained (Figure 1B). Integrating with previous results indicating that starvation can result in the shrinkage of E. coli cells (Shi et al., 2021), E. faecalis incubated in BHI medium for 48 h was chosen as the starvation model for this research.

### 3.2 Effects of iron supply on the survival and biofilm formation of *E. faecalis* under starvation conditions

**Figure 2:**
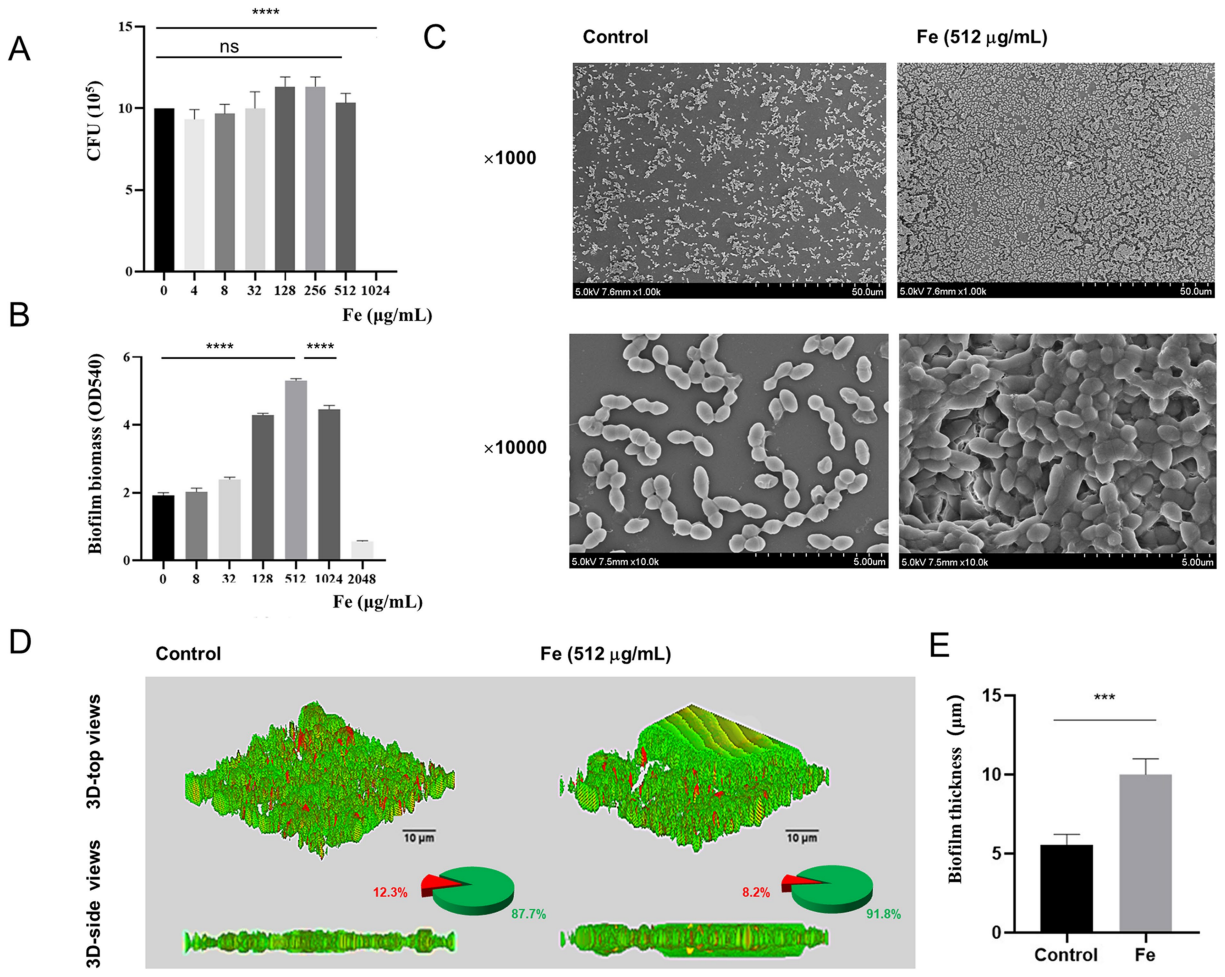
Effects of iron supply on the survival and biofilm formation of starved *E. faecalis*. **(A)** Results of CFU counts of starved *E. faecalis* cultured under different concentrations of Fe salts for 24 h. **(B)** Crystal violet staining results of starved *E. faecalis* biofilms exposed to different concentrations of iron. **(C)** SEM images of starved *E. faecalis* biofilm formed in BHI (Control) or with Fe salt (512 μg/mL). **(D)** LIVE/DEAD staining images, pie charts, and biofilm thickness images of starved *E. faecalis* biofilms after different treatments. Scale bars = 10 μm. **(E)** Statistical plot of biofilm thickness under different treatments. ***p < 0.001, ****p < 0.0001.

Initially, we assessed the influence of varying concentrations of Fe salt on the viability of planktonic *E. faecalis* through CFU counting. When the concentration of Fe salt is below 512 μg/mL, bacterial growth remains essentially unaffected (*p >* 0.05) (Figure 2A). After 48 h of biofilm development, the quantity of biofilms was evaluated by crystal violet staining and measuring OD values at 540 nm, and it was found that the formation of biofilms increased with increasing Fe concentration. Notably, a significant inhibitory effect was only observed when the concentration reached 2048 μg/mL (*p* < 0.05), with 512 μg/mL Fe salt being the optimal concentration for promoting biofilm formation (Figure 2B). Therefore, a final concentration of 512 μg/mL Fe salt was used for subsequent biofilm experiments. SEM images revealed that the control biofilm displayed sparsely distributed bacteria, whereas the Fe-supplement biofilm showed densely packed bacterial clusters with significantly tighter intercellular connections, forming a denser and more robust three-dimensional architecture (Figure 2C). Figure 2D displays the 3D LIVE/DEAD staining CLSM images, pie charts of the ratio of live/dead bacteria, the corresponding biofilm thickness following different treatments. Green color represents live bacteria, red color represents dead bacteria, and yellow color indicates the co-presence of both live and dead bacteria in a particular area. In the control group, the biofilm mainly showed green fluorescence, interspersed with red fluorescent patches. The corresponding pie chart data showed that there was a certain proportion of dead bacteria in the biofilm of this group. The green fluorescence of biofilm in Fe-supplement group was more significant, and the pie chart showed that the proportion of viable bacteria in iron supplementation group was higher than that in control group. The 3D-side view images, along with the bar graph, clearly demonstrated that the biofilm in the Fe-supplement group was thicker than that in the control group (Figure 2E, *p* < 0.05). These findings indicate that the addition of iron had a significant impact on increasing the biofilm thickness, likely promoting bacterial growth and biofilm development.

### 3.3 Effect of interfering with iron supply on biofilm formation of starved *E. faecalis*

**Figure 3:**
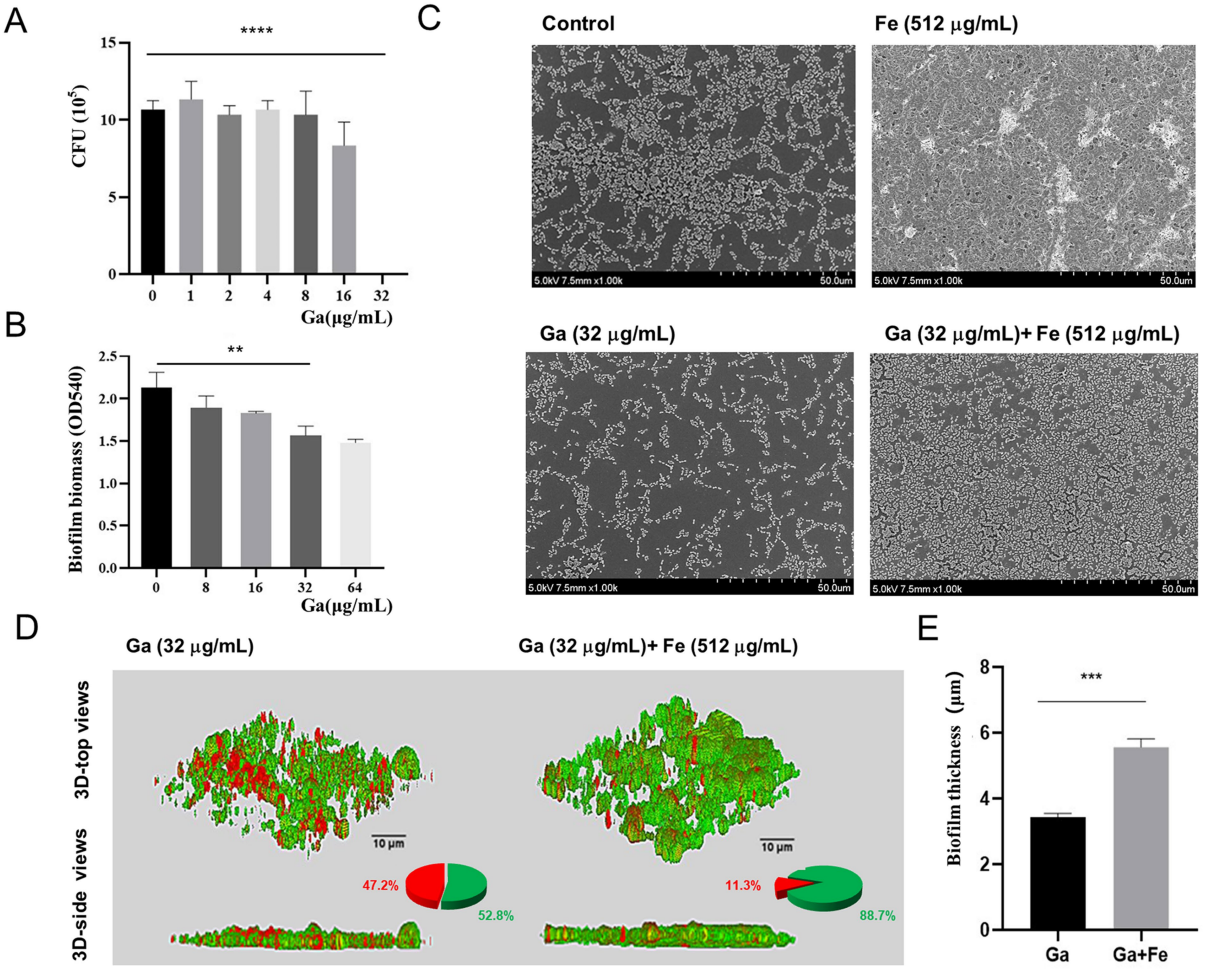
Effect of interfering with iron supply on biofilm formation of starved *E. faecalis*. **(A)** Results of CFU counts of starved *E. faecalis* cultured under different concentrations of Ga salts for 24 h. **(B)** Crystal violet staining results of starved *E. faecalis* biofilms exposed to different concentrations of Ga. **(C)** SEM images of starved *E. faecalis* biofilms under different treatments. **(D)** LIVE/DEAD staining images, pie charts, and biofilm thickness images of starved *E. faecalis* biofilms after different treatments. Scale bars = 10 μm. **(E)** Statistical plot of biofilm thickness under different treatments. **p < 0.01, ***p < 0.001, ****p < 0.0001.

To further validate the role of iron supply on biofilm formation in starved *E. faecalis*, we employed iron-competition inhibitors for assessment. Gallium (Ga), having an ionic radius comparable to that of iron, can penetrate the cytoplasm via the bacterial iron-uptake mechanism. Once inside, it disrupts the bacteria’s native iron-uptake process, thereby inducing iron starvation within bacterial cells (Owusu et al., 2024). Firstly, we found that 32 μg/mL of Ga salt was effective against *E. faecalis* by means of CFU counting (Figure 3A). Subsequently, crystal violet staining showed that 32 μg/mL Ga salt was effective in inhibiting biofilm formation of starved *E. faecalis* (Figure 3B, *p* < 0.05). Therefore, a final concentration of 32 μg/mL of Ga salt was chosen as the concentration for subsequent experiments. SEM results illustrated that, when compared with the control biofilm, Ga salt was able to disintegrate the biofilm architecture and trigger bacterial lysis and destruction. On the contrary, Fe-Ga mixed group gave rise to an augmentation in both the thickness of the biofilm and the count of bacteria (Figure 3C). CLSM analysis demonstrated that, relative to the mixed group, the Ga-treated group manifested a reduction in green fluorescence and an elevation in red fluorescence within the biofilm. The data presented in the pie chart indicated that a significantly higher proportion of dead bacteria was present in the biofilm of the Ga group. Simultaneously, the thickness of the biofilm in the Ga-treated group was decreased by a factor of 1.6 (Figures 3D,E, *p* < 0.05). Therefore, we can speculate that the disruptive effect of Ga on the iron uptake mechanism of *E. faecalis* depends on the concentration of iron. Iron supply can offset the damage of Ga salt on iron uptake mechanism of *E. faecalis*, thereby promoting its biofilm formation.

### 3.4 Correlation analysis between iron supply and eDNA release from starved *E. faecalis* biofilm

**Figure 4:**
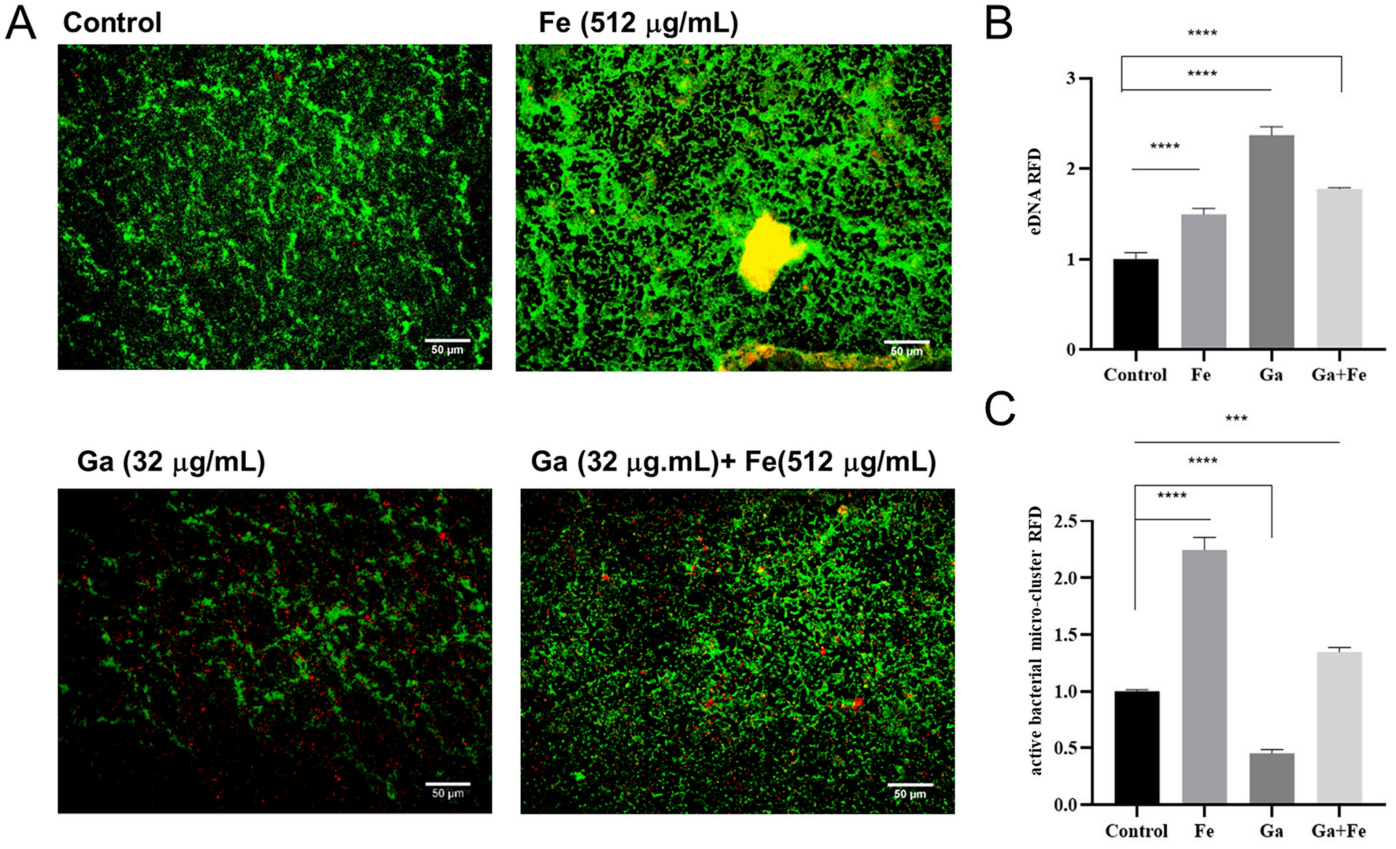
Correlation analysis between iron supply and eDNA release from starved *E. faecalis* biofilms. **(A)** Fluorescence staining results of starved *E. faecalis* biofilms under different treatments. Scale bar = 50 μm. **(B)** RFD of eDNA in biofilms. **(C)** RFD of active bacterial microclusters in biofilms. Results are the mean of one experiment, each of which was repeated three times. ***p < 0.001, ****p < 0.0001.

We then investigated whether eDNA, a key structural component of the biofilm matrix, contributes to the enhanced biofilm formation observed upon iron supply. Fluorescence microscopy and Image J analysis were used to assess the eDNA levels. The fluorescence microscopy results for different treatment groups are depicted in Figure 4. Green fluorescence represents live bacteria, red fluorescence represents eDNA, and the yellow fluorescence resulting from the overlap of red and green fluorescence. In the control group, there was a certain amount of green-fluorescent live bacteria and red-fluorescent eDNA distribution, with some degree of overlap between the two. In the Fe-supply group, the intensity of green and red fluorescence increased significantly, and obvious yellow fluorescence area (overlapping area of viable bacteria and eDNA) appeared, suggesting that eDNA is present in the biofilm rather than within the bacteria. In the Ga-treated group, the fluorescence intensity of green live bacteria decreased, and the red eDNA was distributed more dispersedly. However, the simultaneous addition of Fe notably enhanced the green fluorescence while reducing the red fluorescence. Image J analysis revealed that the relative fluorescence density (RFD) of eDNA in the Fe-supply group was approximately 1.5-fold higher than that in the control group (Figure 4B, *p* < 0.05). Concurrently, the RFD of viable bacterial clusters showed a statistically significant increase in the Fe-supply group compared to the control (Figure 4C, *p* < 0.05). These findings suggest that starved *E. faecalis* cells require elevated iron levels to facilitate biofilm formation. Notably, treatment with Ga salts led to a striking 2.73-fold increase in eDNA relative abundance within the biofilm (Figure 4B, *p* < 0.05), whereas the relative abundance of viable bacterial clusters exhibited a approximately 1-fold reduction (Figure 4C, *p* < 0.05). In the Ga-Fe mixed group, the eDNA RFD value was lower than that observed in the Ga-alone group but remained significantly higher than the control group (*p* < 0.05).

**Figure 5:**
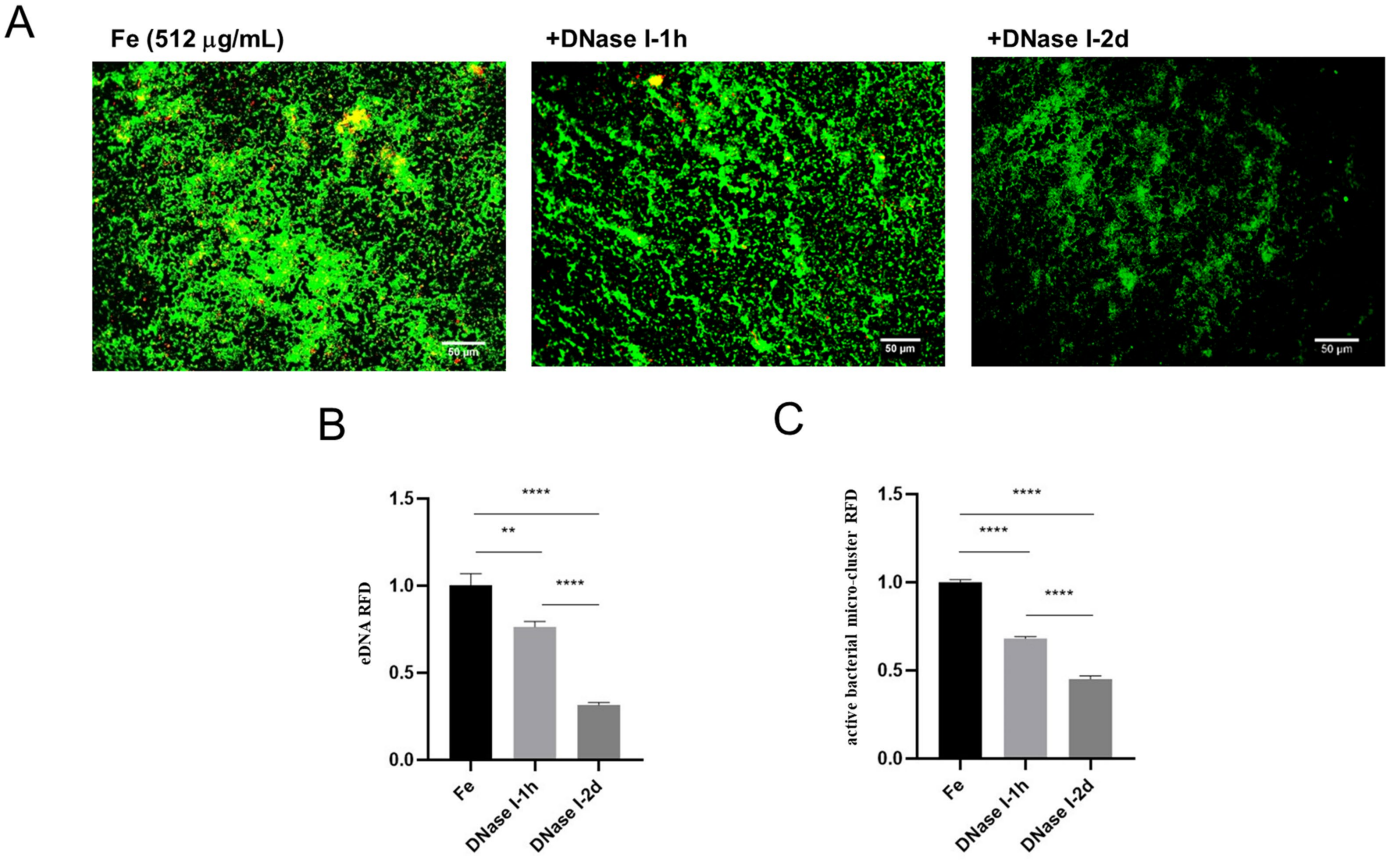
Effect of different DNase I treatment times on starved, Fe-supply *E. faecalis* biofilms. **(A)** Fluorescence staining results of starved *E. faecalis* biofilms under different treatments. Scale bar = 50 μm. **(B)** RFD of eDNA in biofilms. **(C)** RFD of active bacterial microclusters in biofilms. Results are the mean of one experiment, each of which was repeated three times. **p < 0.01, ****p < 0.0001.

In order to further explore the relationship between iron supply and eDNA release, and the effect of this relationship on the biofilm formation of *E. faecalis* under starvation condition, we carried out relevant experiments. Given that previous studies have shown that DNase I is able to inhibit eDNA dependent biofilm formation (Blakeman et al., 2019), this study focused on the effects of DNase I treatment on the parameters associated with iron-treated starved *E. faecalis* biofilms. The experimental results were presented by fluorescence microscopy images and quantitative analysis data. In the images of the Fe-supply group, a large number of live bacteria exhibiting green fluorescence and a certain amount of eDNA showing red fluorescence were observed, with some yellow overlapping regions between them. This clearly indicates the presence of a substantial number of live bacteria and a significant quantity of eDNA within the biofilm. After DNase I treatment for 1 h in the iron-supply group, the green and red fluorescence intensities were significantly attenuated compared to the untreated iron-supply control, and the yellow overlapping area was also reduced. In the DNase I treated 2 d group, the fluorescence intensity of green live bacteria was significantly attenuated, the fluorescence intensity of red eDNA was further decreased, and the overall brightness of the image was markedly dimmed. This implies that after 2 d of DNase I treatment, both the eDNA amount and the number of live bacteria in the biofilm decreased significantly (Figure 5A). In the quantitative analyses presented in panels B and C, when the Fe-supply samples were subjected to DNase I treatment, as the treatment time extended from 1 h to 2 d, the RFD of both the micro-clusters (Figure 5B) and the eDNA (Figure 5C) in the biofilm demonstrated a significant downward trend (*p* < 0.05). These results indicate that DNase I exerted a significant impact on both live bacteria and eDNA in the iron-supplied biofilms, effectively reducing the relative fluorescence densities of both. Moreover, the effect of the 2 d treatment was significantly more pronounced than that of the 1 h treatment. This phenomenon implies that iron supply may promote the biofilm formation of starved *E. faecalis* by regulating the release of eDNA.

## 4 Discussion

*Enterococcus faecalis* is the most frequent species present in post-treatment disease and plays a significant role in persistent periapical infections following root canal treatment (Zilm et al., 2017). After a series of root canal therapy steps such as root canal disinfection and root canal preparation, conditions of starvation and alkalinity provide a harsh living environment for *E. faecalis*, but these microorganisms can tolerate stress conditions under alkaline and glucose starvation stress (Chen et al., 2017). It has been confirmed that this bacterium adapts its transcriptome under stress conditions to colonize the host, without increasing its virulence but favoring its pathogenesis and survival (Salze et al., 2020). This favors the survival of the bacterium in the obturated root canal and it can regain the ability to multiply with serum-derived nutrients, thus having the potential to generate periapical lesions (Richards et al., 2010). Therefore, in order to simulate the conditions faced by *E. faecalis* in the root canal system, this study monitored the growth curve of *E. faecalis* without addition of new nutrients as described by Portenier et al. (2005). The results demonstrated that the activity of *E. faecalis* declined significantly following 24 h of incubation and reached a stable state after 48 h of incubation. Subsequently, SEM analysis of the bacteria incubated for 48 h revealed morphological changes characteristic of a starvation state. The observed cell shrinkage was consistent with previous findings on starved *Escherichia coli*, further validating the presence of this starvation-related phenotype. Therefore, for this experiment, we selected *E. faecalis* cultured in the medium for 48 h as the starvation – state bacterial model.

Iron is an essential element for bacterial growth and metabolism, and an indispensable cofactor of numerous bacterial biologic processes (Sheldon et al., 2015). For example, Keogh reported a novel form of iron-dependent metabolism of *E. faecalis* in absence of heme, in which iron can participate in extracellular electron transfer to promote biofilm growth and alter the biofilm matrix (Keogh et al., 2018). And Deena suggested that iron supply increased the antibiotic resistance of *E. faecalis* in both single- and dual-species bacterial cultures (Govindarajan et al., 2022). In this study, we found that iron supply also contributed to the biofilm formation of starved *E. faecalis* inducing the transformation of flat biofilms into three-dimensional biofilms. To further analyze the effect of iron supply on starved *E. faecalis* biofilms, Ga^3+^, whose ionic radius and binding tendency are very similar to Fe^3+^, was cited for analysis, which can act as an iron analog to bind Fe^3+^-bound compounds, such as heme transport systems, leading to bacterial iron starvation, which is known as a “Trojan horse” strategy (De Leseleuc et al., 2012). At present, simple Ga salt such as gallium nitrate have been approved by the FDA for the treatment of clinical hypercalcemia (Song et al., 2019). The findings of our study demonstrate that Ga salts, by interfering with iron supply, can inhibit the biofilm formation of starved *E. faecalis*. Notably, the results obtained from SEM and CLSM in the mixed group reveal that environmental iron can, to a certain degree, mitigate the disruption of biofilm formation induced by Ga salts. This result indicates that the availability of iron in the environment exerts a pivotal influence on the biofilm formation process of starved *E. faecalis*.

The eDNA plays an important role in biofilm formation (Gloag et al., 2013). It has been reported that iron strongly affect bacterial lysis and eDNA release during oneidensis biofilm formation (Binnenkade et al., 2014). Based on these findings, we investigated the correlation between iron supply and eDNA release in promoting the biofilm formation of starved *E. faecalis* by fluorescent staining, and the results showed that active bacterial microclusters and eDNA were increased in Fe-supply group. These data suggests that iron supply and eDNA release might play synergistic roles in promoting the biofilm formation of starved *E. faecalis*. Subsequently, we verified the changes of eDNA and active bacteria in biofilms by interfering with bacterial iron supply by Ga salt. Results show that the Ga-treated group has more eDNA than the Fe and Ga-Fe mixed groups, yet its biofilm mass is lower. In line with our findings, previous research demonstrated that gallium nitrate exerted similar effects on *Staphylococcus aureus* biofilms (Xia et al., 2021), thereby validating the generality of this phenomenon. Moreover, our prior study identified that Ga salt at a final concentration of 32 μg/mL inhibited the growth of *E. faecalis.* Differently, in the mixed group, the fluorescence intensities representing eDNA and viable bacteria were between those of the Fe-supply group and the Ga-treated group. This is most likely due to the competitive inhibition between Fe and Ga (De Leseleuc et al., 2012). Such inhibition attenuates the individual effects of Fe and Ga on the biofilm. Collectively, these findings demonstrate that the extent of biofilm formation and eDNA release by starved *E. faecalis* varies significantly with iron availability. Subsequently, we found that DNase I treatment was capable of effectively diminishing both the eDNA content and the number of viable bacteria within the iron-supplied biofilms. As the treatment time was prolonged from 1 h to 2 d, not only did the eDNA content decline substantially, but the quantity of viable bacteria in the biofilm was also significantly impacted. These strongly suggest that iron supply may affect biofilm construction in starved *E. faecalis* by regulating eDNA release.

Although similar results have been reported previously, those studies only showed that the effect of Fe deficiency on biofilm formation was dependent on eDNA release (Qiao et al., 2018). Here, we demonstrated for the first time that iron supply may promote active bacterial proliferation by regulating the release of eDNA, thereby promoting biofilm formation in starved *E. faecalis*. This study has several limitations that should be considered when interpreting the findings. Firstly, for the mechanism by which iron supply promotes biofilm formation in starved *E. faecalis*, although a hypothesis related to increased eDNA release has been proposed, further in-depth studies are needed to clarify the specific molecular mechanisms and signaling pathways. Second, the experiment only used specific gallium preparations and monitoring methods, which may have some limitations, and more different experimental means and reagents are needed for verification and supplement. Thirdly, the performance and impact of starvation *E. faecalis* in the actual clinical environment may need to be further analyzed and explored in combination with more clinical cases and actual conditions.

## 5 Conclusion

Iron supply potentially promotes the active proliferation of bacteria by regulating the release of eDNA, thereby facilitating the biofilm formation of starved *E. faecalis*. This research offers novel insights and evidence for a deeper understanding of the mechanism underlying the biofilm formation of *E. faecalis* under starvation conditions. In future investigations and control strategies targeting *E. faecalis* biofilm formation, it is essential to take into account the combined effects of iron and other environmental factors.

## Data Availability

The raw data supporting the conclusions of this article will be made available by the authors, without undue reservation.
